# Supplementary material for: The impact of mental and somatic stressors on physical activity and sedentary behaviour in adults with type 2 diabetes mellitus: a diary study
Source: PeerJ. 2021 Jun 18;9:e11579. doi: 10.7717/peerj.11579 (PMC8216170; doi:10.7717/peerj.11579)
Supplement: Supplemental Information 1 [file peerj-09-11579-s001.docx]

Characteristics of participants who did (not) provide valid accelerometer and diary data

| **Characteristics** | **Included (N = 39)** | **Excluded (N = 15)** |
| --- | --- | --- |
| Sex  N women (%) | 13 (33%) | 7 (47%) |
| Age in years  mean (SD); range | 63.18 (7.80); 50.00-81.00 | 61.18 (9.09);  46.00-77.00 |
| Educational level  N high level of education (%) | 20 (51%) | 7 (46%) |
| Retirement  N retired (%) | 19 (49%) | 7 (46%) |
| Waist circumference in cm  mean (SD); range | 109.28 (14.94); 75.55-155.75 | 109.10 (12.03);  80.80-133.00 |
| BMI (kg/m²)  mean (SD); range | 30.82 (6.00);  21.40-50.90 | 31.49 (4.57);  25.65-42.11 |
| Time since diagnosis in months  mean (SD); range | 129.40 (83.31);  12.00-288.00 | 130.70 (85.64);  12.00-300.00 |
| Level of anxiety  mean (SD); range | 10.08 (4.02); 6.00-24.00 | 10.33 (3.66) 6.00-17.00 |
| Level of depression  mean (SD); range | 8.56 (3.65); 6.00-23.00 | 10.07 (6.43); 6.00-30.00 |
