# Supplementary material for: The impact of mental and somatic stressors on physical activity and sedentary behaviour in adults with type 2 diabetes mellitus: a diary study
Source: PeerJ. 2021 Jun 18;9:e11579. doi: 10.7717/peerj.11579 (PMC8216170; doi:10.7717/peerj.11579)

Individual responses for the mental and somatic stressors

# Fatigue

#
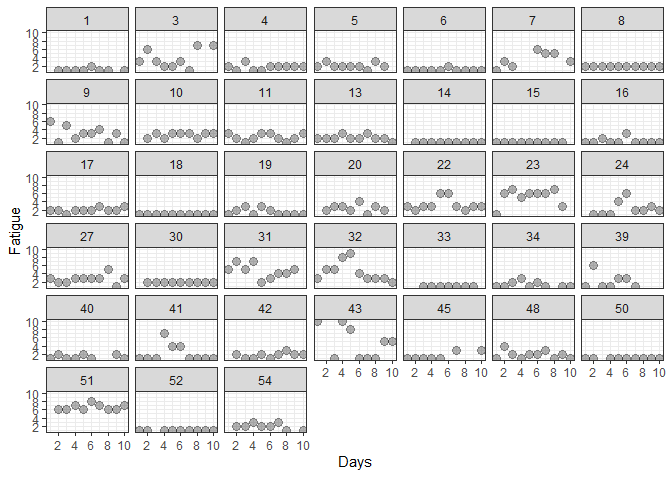


# Stress


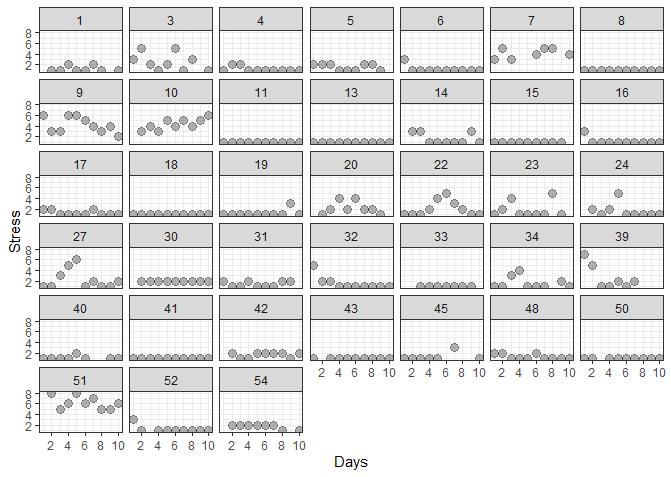


# Pain

#
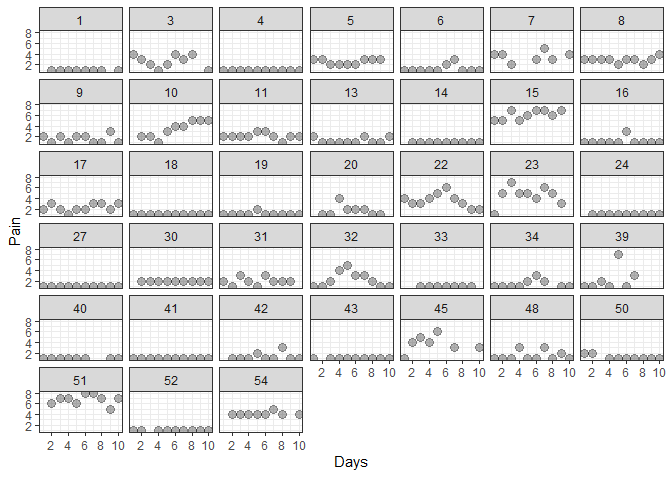


# Nausea/dizziness

#
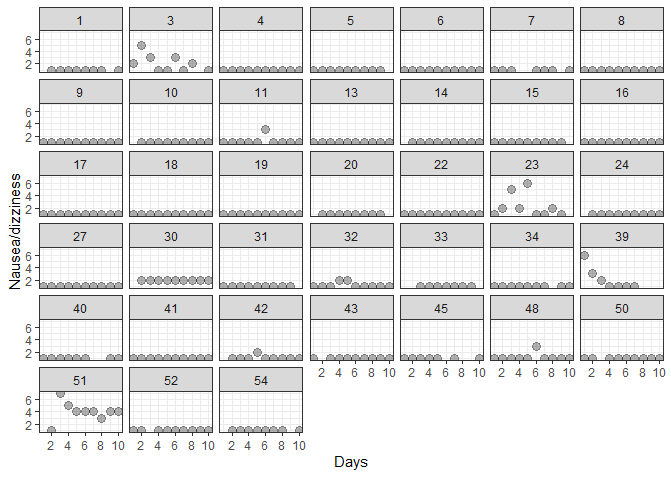


# Numbness/tingling


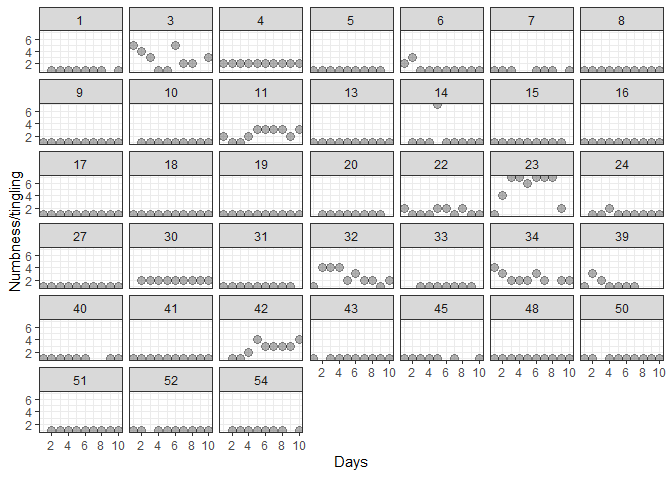


# Sadness


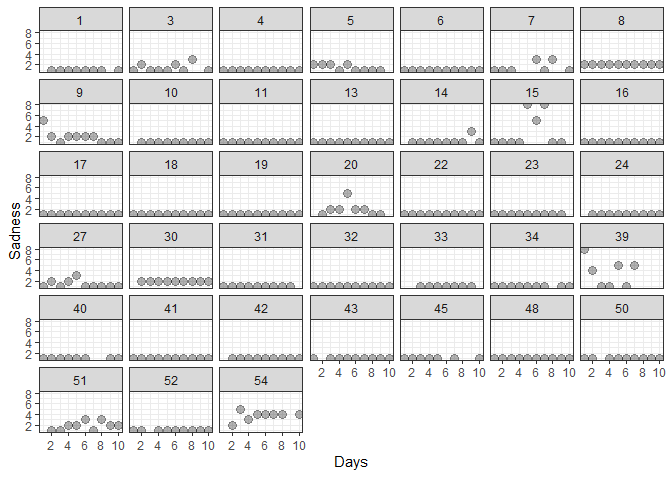

Supplement: Supplemental Information 2 [file peerj-09-11579-s002.docx]
